# Influence of Piau and commercial genetic groups on the performance, carcass traits, and meat quality of pigs

**DOI:** 10.1007/s11250-026-05335-9

**Published:** 2026-09-21

**Authors:** Layla Cristien de Cássia Miranda Dias, Paulo Sávio Lopes, Ricardo Martin da Silva Bernardo, Caroline Pereira Abreu, Guilherme Henrique de Freitas, Mario Luiz Chizzotti, Delvan Alves da Silva, Daniele Botelho Diniz Marques, Renata Veroneze

**Affiliations:** Department of Animal Science, Universidade Federal de Viçosa, Avenida PH Rolfs, s/nº, Campus Universitário, Viçosa, CEP: 36570- 900 MG Brazil

**Keywords:** Crossbreeding, Sustainability, Local breed, Genetic resources

## Abstract

The Piau is a naturalized Brazilian pig breed known for its disease resilience, low maintenance and nutritional requirements, and adaptability to diverse climatic conditions. Incorporating local breeds, such as Piau, into crossbreeding programs with commercial lineages may increase the productive performance of naturalized breeds while helping preserve their genetic diversity. This study evaluated the influence of genetic groups on the performance, carcass traits, and meat quality of barrows. Thirty-nine animals from four groups: purebred Piau (PP), Large White (LL), Piau × Large White (PL), and Duroc × Large White (DL) were analyzed in a completely randomized design. Performance, carcass yield, chemical composition, and meat quality (*Longissimus dorsi* and *Biceps femoris*) were assessed. Results showed that PP had the lowest growth rates, while DL exhibited the highest productive efficiency. The PL crossbreed showed significantly higher hot carcass weight compared to PP, reaching levels near those of commercial groups while retaining the high backfat thickness characteristic of the Piau breed. Regarding meat quality, PP and PL showed superior water-holding capacity and lower drip loss compared to commercial lines. Although shear force was similar for the loin across groups, DL had the most tender ham (*Biceps femoris*), while PL showed lower tenderness for this cut. In conclusion, crossbreeding Piau with Large White (PL) increases performance and carcass weight in comparison to purebred Piau without compromising the distinctive moisture retention and fat attributes of the local breed. The highest productive performance was observed for the Duroc × Large White cross.

**Supplementary Information:**

The online version contains supplementary material available at https://doi.org/10.1007/s11250-026-05335-9.

## Introduction

Pig production is of significant economic and social importance in Brazil, contributing to the country’s status as one of the world’s leading pork producers (ABPA 2025). Pork serves as a vital protein source for human nutrition, particularly in less developed countries. In response to the growing demand for high-quality meat, the sector has increasingly adopted new technologies, particularly those related to genetic improvement and high-productivity systems. These advancements, when integrated with sustainable practices, not only enhance pork production and quality but also lead to an increase in its market value (Rushchitskaya et al. 2024).

Brazilian pig farming has evolved from a primarily subsistence activity into an advanced, large-scale sector (Callegari et al. 2020). This transformation has been driven by the adoption of intensive production systems, resulting in the widespread use of breeds with high genetic potential. These breeds were selected for increased meat production, precocity, prolificacy, feed conversion, growth, and high carcass yields. Consequently, the use of local Brazilian breeds has declined due to the preference for genetically improved commercial breeds (Mariante et al. 2003).

Distinct pig breeds play specific roles in global and national production systems. The Large White breed is widely favored globally for its exceptional prolificacy, rapid growth rates, and high lean meat yield, making it highly efficient in intensive production systems (Rosendo et al. 2012; Saikia et al. 2024). The Duroc breed is also a common choice, particularly valued as a terminal sire for its superior meat quality attributes, including higher intramuscular fat content, enhanced tenderness, and robust growth performance (Suzuki et al. 2005a, b. The lipid profile of Duroc, particularly its intramuscular fat, makes it highly desirable for the production of high-value processed products, such as dry-cured hams (Ruiz et al. 2002).

Local Brazilian breeds, such as the Piau, exhibit slower growth rates and higher total fat deposition, particularly subcutaneous fat, compared to modern genotypes. The Piau is one of the most important local pig breeds in Brazil, known for its disease resilience, low maintenance and nutritional requirements, and its ability to adapt to diverse Brazilian climatic conditions (Sollero et al. 2009; Veroneze et al. 2014; Bulos et al. 2016). Evaluating the production potential and meat attributes of local Brazilian pig breeds is essential for its conservation and inclusion in the pork production chain.

While the pursuit of high productivity and efficiency in intensive systems yields undeniable economic benefits, it can also lead to significant trade-offs in meat quality traits. Genetic improvement has primarily focused on weight gain, feed conversion, and lean carcass traits, frequently neglecting meat quality attributes such as flavor, juiciness, tenderness, and intramuscular fat content, which are critical to the consumer’s sensory experience (Hoa et al. 2020). Despite their superior performance in production parameters, commercial breeds may present challenges regarding meat quality traits and adaptability to less intensive systems or adverse environmental conditions.

To leverage the strengths of diverse genotypes and enhance both efficiency and product quality, crossbreeding is widely utilized in pig production (Zhang et al. 2018). This strategy exploits heterosis and breed complementarity, enabling the integration of desirable traits from distinct genetic lines. Incorporating local breeds, such as Piau, into crossbreeding programs with commercial lineages may increase the productive performance of naturalized breeds while helping preserve their genetic diversity. These breeds have also been highlighted for their potential socioeconomic role in smallholder systems (Araújo et al. 2020). Population numbers of the Piau breed have declined in recent decades (Veroneze et al. 2014), underscoring the need for a better understanding of its production potential. Therefore, to assess the impact of genetic group on performance, carcass traits, meat quality, and meat chemical composition, we evaluated castrated males from four genetic groups Piau (PP), Large White (LL), Piau x Large White (PL), and Duroc x Large White (DL).

## Materials and methods

All methods involving animals used in this study agree with the National Council for Animal Experiment Control (CONCEA) and were approved by the Ethics Committee on Animal Use Production (CEUAP-UFV, nº14/2022).

### Animals, housing and experimental design

Thirty-nine growing barrows were used from the Swine Breeding Teaching, Research, and Extension Unit at Universidade Federal de Viçosa (UFV), Minas Gerais, Brazil. Animals were from different genetic groups, with six animals of the local Brazilian breed Piau (PP, *n* = 6), nine Large White (LL, *n* = 9), eleven crossbred Piau x Large White (PL, *n* = 11), and thirteen crossbred Duroc x Large White (DL, *n* = 13). The Piau × Duroc crossbreeding was not performed due to the unavailability of Duroc females at the time of the study. Pigs from all genetic groups were randomly allocated into individual concrete pens (2.41 × 1.36 m) at 70.79 ± 1.88 days of age, with free access to water and feed. The first seven days were considered the adaptation period. Therefore, the experiment was initiated when the animals reached 77.79 ± 1.88 days of age. The experimental design was completely randomized, and each animal corresponded to an experimental unit. The diets were formulated according to the recommendations of Rostagno et al. (2017) to meet the animals’ nutritional requirements. The performance trial consisted of four feeding phases, with identical rations formulated according to the specific requirements of each life stage and administered consistently across all genetic groups. The growth phases and feed changes were defined based on the age of the animals (in days) as follows: growth stage 1 (G1), 77 to 98 days; growth stage 2 (G2), 99 to 119 days; finishing stage 1 (F1), 120 to 140 days; and finishing stage 2 (F2), 141 to 165 days of age (Supplementary Table 1). Due to operational issues, slaughter took place 12 days after the end of the finishing phase 2 (F2).

Body weights were recorded using a digital scale at the onset of the experiment (day 0) and upon the completion of each feeding phase (G1, G2, F1, and F2); weighing was consistently performed without prior fasting. To ensure precise data collection, both the total feed supplied and the individual orts remaining at the end of each phase were weighed. These records, integrated with the body weight data, allowed for the assessment of performance parameters for each phase, including average daily feed intake (ADFI), average daily gain (ADG), and feed conversion ratio (FCR).

### Slaughter, carcass yield and primal cuts

Pigs were slaughtered at 177.28 ± 2.10 days of age, following a sixteen-hour fast. Each animal was weighed the day before slaughter to determine the final slaughter weight (SW). After slaughter, carcasses were divided into longitudinal halves, identified, and weighed to obtain the hot carcass weight (HCW). Subsequently, the carcasses were chilled at 4 °C for 24 h and weighed to determine the cold carcass weight (CCW). Hot and cold carcass yields (HCY and CCY, respectively) were calculated as the percentage of the respective carcass weight relative to the final SW.

Loin eye area (LEA) and backfat thickness (BT) were measured on the left side of the cold carcass. To facilitate these measurements, a transverse cut was made between the 12th and 13th thoracic vertebrae to expose the *Longissimus dorsi* muscle. The muscle perimeter was traced onto an acetate sheet, and the LEA was subsequently estimated by placing the tracing over a standardized grid and counting the squares within the area. At the same cross-section, BT was measured over the *Longissimus dorsi* muscle using a digital caliper.

Also, in the center of the *Longissimus dorsi* muscle on the left half-carcass, between the 12th and 13th thoracic vertebrae, pH was measured at three time points: immediately post-slaughter (pH0), 45 min post-slaughter (pH45), and 24 h after post-mortem chilling (pH24). A pH meter (Hanna-DIGIMED model DM-20) equipped with a penetration probe (DIGIMED, DME-CV1) was used for pH readings. Carcass temperature was simultaneously recorded at the same location using a digital probe thermometer (or digital immersion/penetration thermometer) equipped with a stainless steel pointed probe, ensuring accurate internal temperature readings from the core of the muscle.

From the right carcass primal cuts were obtained: head; sirloin; loin with tenderloin, sirloin, fat and skin (WL); rib with fat, skin and bone (WR); belly; ham with fat, skin and bone (WH); picnic shoulder with fat, skin and bone (WS); and feet. Subsequently, the skin and fat were removed, and the loin, ham, rib and picnic shoulder were weighed again. Also, from the loin the cuts of sirloin and tenderloin were obtained. Cut yields were calculated as the proportion of each cut’s weight to the right cold half-carcass weight, expressed as a percentage. From the left half-carcass, a complete dissection was performed, where lean meat, fat, and bones were manually separated and individually weighed.

All cuts were performed according to technical specifications to ensure standardization (ABCS 2010). The ham was obtained from the posterior portion by separation at the coxofemoral joint, including the pelvic bones and femur. The picnic shoulder comprised the distal scapula and humerus region, while the sirloin was obtained from the cranial-dorsal portion (1st to 4th thoracic vertebrae and adjacent muscles). The loin was located dorsal to the belly, extending posteriorly from the sirloin to the ham. The belly encompassed the ventral abdominal region, from the 5th rib to the pubic symphysis, including the abdominal muscles and ventral rib segments. The ribs included the thoracic region from the 5th to the 13th rib, along with the intercostal muscles and costal arches.

### Meat quality analysis

Drip loss was determined for the loin (*Longissimus dorsi*) and ham (*Biceps femoris*) samples according to the method described by (Honikel 1987). The samples were collected 24 h after slaughter, and the visible connective tissue and fat tissues were removed. For this analysis, the *Longissimus* samples were specifically collected from the 14th rib and then cut into slices weighing 8.387 g ± 3.892 g. The *Biceps femoris* samples were cut into slices weighing 7.143 g ± 3.531 g. Each slice was placed in an airtight container and suspended at 4 °C. After a 48-hour refrigeration period, the samples were reweighed. Drip loss was calculated as the difference between the initial and final weights, expressed as a percentage of the initial weight.

Steaks from *Biceps femoris* and the *Longissimus dorsi* were used for color measuarement using a previously calibrated Hunter MiniScan EZ (4500 L, Hunter Associates Laboratory, Inc., Reston, Virginia, USA). Five scanning were taken at various positions on the surface of the lean meat in each steak to determine the values of L* (lightness), a* (redness), and b* (yellowness). These L*, a* and b* parameters describe meat appearance, with L* indicating lightness, ranging from 0 for black to 100 for white; a* indicating redness, positive values signify more red, while negative values signify greenness, and b* indicating yellowness, positive values signify more yellow, while negative values signify blue. Average of the five measurements made on the lean meat and fat for both muscles was used to obtain the final values for each parameter.

Thawing loss, cooking loss, and Warner-Bratzler shear force (WBSF) were determined for both muscles. Samples were weighed before and after thawing to calculate the amount of water lost during the process (thawing loss). Thawed steaks were vacuum-packed and cooked at 70 °C for 40 min in a thermostatically controlled water bath (Bruce et al. 2004). Cooking loss was determined by the weight difference before and after cooking, while total loss was calculated based on the difference between the initial frozen weight and the final cooked weight. All losses were expressed as a percentage of the initial sample weight.

After cooking, steaks were allowed to cool for 30 min in running water (approximately 20 °C). After cooling, each steak had seven circular cores (1.27 cm in diameter) taken out of it in parallel to the long axis of the muscle fibers (American Meat Science Association, 1995). A Warner-Bratzler shear machine (G-R Manufacturing Company, Manhattan, KS, USA) outfitted with a 1.016 mm thick V-shaped blade was used to shear each core once through the center and perpendicular to the direction of the fibers at a constant speed of 2 mm/s.

### Chemical composition analysis

The analyses carried out for the preparation and determination of the dry matter, mineral matter and crude protein contents were carried out following protocols described by the Methods for Food Analysis of the National Institute of Science and Technology in Animal Science (Detmann et al. 2021), and were carried out at the Animal Nutrition Laboratory (UFV). The determination of intramuscular fat content, ether extract (EE), was carried out according to the method prescribed by the Association of Official Analytical Chemists (AOCS, 2009).

The natural matter was measured beforehand, and the samples were subjected to the freeze-drying process according to method G-001/2 (Detmann et al. 2021) to reduce the moisture content in an oven with forced air circulation. The final dryness and dry matter content of the samples were determined using method G-003/1, for oven drying without forced air ventilation, as set out in Detmann et al. (2021), and the mineral matter, crude protein and ether extract contents were expressed based on dry matter.

The mineral matter content in the samples was measured using the muffle furnace method, method M-001/2 (Detmann et al. 2021). The process involved a gradual heating process, starting at 125 °C, then increasing to 250 °C and finally to 350 °C, with a 30-minute stabilization period at each temperature. Subsequently, the temperature was raised to 550 °C for the designated burning time as per the method.

The crude protein content was determined using the Kjeldahl method described by Detmann et al. (2021), according to method N-001/2. Due to the nitrogen content present in the meat samples, 150 mg of sample was weighed for each aliquot and titration was carried out with a strong acid solution (hydrochloric acid (0.05 N), factor 0.9921 and correction 69.44). The salt used in the digestion mixture was potassium sulfate (K2SO4). A heating ramp was also carried out during the digestion stage. The ramp started at 150 °C, and the temperature was increased by 50 °C every 30 min until the temperature designated by the method adopted was reached.

### Collagen content determination

Collagen content was determined based on a methodology developed by (Cross et al. 1973) and refined by Latorre et al. (2004) and Hadlich et al. (2006). Total collagen (mg/g) and soluble collagen (% of the total) were measured using their hydroxyproline content. Five grams of homogenized steak were taken, put in plastic tubes with 20 mL of distilled water, and then heated in a water bath for two hours at 80 °C. Following cooking, the samples were centrifuged at 1000 g for 15 min at room temperature, then homogenized for 1 min in a Turrax mixer. Aliquots of 30 and 50 mL oft 6 M HCl were added to the supernatant and sediment fractions, respectively, after they had been separated into glass tubes. For hydrolysis, the samples were heated to 100 °C in a dry oven for 16 h. Following filtering, the samples were divided into 1:10 and 1:25, and the pH was then raised to 6.0 using 2 M NaOH. Following this step, 2 mL each of the supernatant and sediment fractions as well as 1 mL of the oxidizing solution (10 mL of Chloramine solution T 7% and 40 mL of Citrate Buffer) were transferred to test tubes. After 20 min at room temperature 1 mL of the colorant (5 g of 4-dimethylaminobenzaldehyde, 20 mL of propanol, and 9 mL of 60% perchloric acid) were added. The samples were kept in a water bath at 60 °C for 15 min. Their absorbance at 560 nm was determined after cooling.

### Statistical analysis

Growth performance, carcass traits, meat quality, chemical composition and collagen content measurements were analyzed using a completely randomized design (CRD), where each animal was considered an experimental unit. The data were subjected to analysis of variance (ANOVA) according to the following model:

$$\:{y}_{ij}=\:\mu\:+\:{g}_{i}+\:{e}_{ij}$$

Where $\:{y}_{ij}$ refers to response variable is the i-th level effect of genetic group, $\:\mu\:$ is constant, $\:{g}_{i}$ is the i-th genetic group and $\:{e}_{ij}$ random error associated with $\:{y}_{ij}$. All analyses were performed using *car* and *emmeans* R packages (R Development Core Team, Vienna, AT). The comparison of the four genetic groups was carried out using Tukey’s test, and differences between means were considered significant at *P* ≤ 0.05.

## Results

### Performance

The PP group showed significantly lower feed intake, average daily gain (ADG), and body weight (BW) across all experimental phases compared with the other genetic groups (*P* < 0.05; Table 1). The PL group exhibited superior BW and improved FC at the end of each phase compared to the purebred PP group. The DL crossbreed positively outperformed all other groups, including the purebred LL. In F2, the DL group reached the highest BW (149.00 ± 8.993 kg) and the best FC (3.770 ± 0.341 kg/kg; *P* < 0.05). During Phase 1 (F1), no significant differences in FC were observed among the PP, PL, and LL groups (*P* > 0.05), while the DL group remained the most efficient (*P* < 0.05). Also on F1, PL did not differ from LL regarding ADG and ADFI. The PP showed lower feed intake, average daily gain, and body weight in all phases than the commercial breeds and crosses (*P* < 0.05). However, the PL crossbreed showed higher weight at the end of the phases and lower feed conversion when compared to the PP group (Table 1).

**Table 1:** Means and standard deviations of performance traits for pig genetic groups from 77 to 165 days of age

Phase	Trait	Genetic group				P-value*
		DL	LL	PL	PP	
G1	BW (kg)	60.800 ± 5.672^a^	54.100 ± 4.569^b^	49.200 ± 3.080^b^	31.800 ± 3.344^c^	< 0.001
	ADFI (kg)	2.390 ± 0.341^a^	2.110 ± 0.250^ab^	2.150 ± 0.241^a^	1.760 ± 0.233^b^	< 0.001
	ADG (kg)	1.130 ± 0.129^a^	0.937 ± 0.092^b^	0.834 ± 0.085^b^	0.582 ± 0.040^c^	< 0.001
	FC (kg/kg)	2.110 ± 0.165^c^	2.260 ± 0.235^c^	2.580 ± 0.223^b^	3.020 ± 0.459^a^	< 0.001
G2	BW (kg)	88.600 ± 6.997^a^	78.70 ± 7.738^b^	69.20 ± 4.165^c^	46.80 ± 4.353^d^	< 0.001
	ADFI (kg)	3.030 ± 0.536^a^	2.810 ± 0.365^a^	2.650 ± 0.325^a^	1.990 ± 0.348^b^	< 0.001
	ADG (kg)	1.322 ± 0.162^a^	1.175 ± 0.164^a^	0.954 ± 0.078^b^	0.716 ± 0.066^c^	< 0.001
	FC (kg/kg)	2.300 ± 0.414^b^	2.410 ± 0.245^ab^	2.790 ± 0.382^a^	2.780 ± 0.352^ab^	< 0.001
F1	BW (kg)	114.700 ± 7.201^a^	99.100 ± 8.800^b^	88.100 ± 5.073^c^	61.700 ± 4.516^d^	< 0.001
	ADFI (kg)	3.410 ± 0.252^a^	3.140 ± 0.243^ab^	2.960 ± 0.336^b^	2.560 ± 0.306^c^	< 0.001
	ADG (kg)	1.244 ± 0.113^a^	0.968 ± 0.099^b^	0.902 ± 0.084^b^	0.706 ± 0.068^c^	< 0.001
	FC (kg/kg)	2.760 ± 0.226^b^	3.260 ± 0.266^a^	3.290 ± 0.313^a^	3.620 ± 0.178^a^	< 0.001
F2	BW (kg)	149.00 ± 8.993^a^	128.00 ± 8.149^b^	115.00 ± 7.475^c^	80.00 ± 7.576^d^	< 0.001
	ADFI (kg)	3.980 ± 0.354^a^	3.680 ± 0.108^ab^	3.510 ± 0.343^b^	2.730 ± 0.398^c^	< 0.001
	ADG (kg)	1.063 ± 0.124^a^	0.911 ± 0.087^b^	0.831 ± 0.107^b^	0.573 ± 0.103^c^	< 0.001
	FC (kg/kg)	3.770 ± 0.341^c^	4.070 ± 0.391^bc^	4.250 ± 0.342^b^	4.830 ± 0.629^a^	< 0.001

G1:growth stage 1 (77 to 98 days of age); G2: growth stage 2 (99 to 119 days of age); F1: finishing stage 1 (120 to 140 days of age); F2: finishing stage 2 (141 to 165 days of age); BW: body weight at the end of the phase; ADFI: average daily feed intake, ADG: average daily gain and FC: feed conversion, measured at 98, 119, 140 and 165 days of age. SD: standard deviation. *ANOVA P values

### Carcass traits

For carcass traits, the PP group (FBW = 81.10 ± 7.31 Kg, HCW = 64.20 ± 6.27 Kg and CCW = 63.10 ± 6.23 Kg) exhibited lower final body weight (FBW), hot carcass weight (HCW), and cold carcass weight (CCW) compared to the PL, LL, and DL groups, respectively (*P* < 0.05; Table 2). Regarding hot carcass yield (HCY), the PP group (79.50 ± 1.28%) did not differ from the DL (81.00 ± 1.95%) and PL (80.70 ± 0.83%) groups, while the LL (80.00 ± 0.94%) group showed a greater HCY. Furthermore, the PP (32.00 ± 9.13 mm and 27.00 ± 5.19 cm^2^) and PL (36.40 ± 4.75 mm and 36.80 ± 9.56 cm^2^) groups exhibited no significant difference in backfat thickness (BT) and loin eye area (LEA), respectively, with these groups having the greatest BT and the lowest LEA. The PL (93.50 ± 6.25 Kg and 91.60 ± 6.28 Kg, respectively) group demonstrated higher HCW and CCW compared to the PP group.

**Table 2:** Means and standard deviations of carcass traits for barrows from four genetic groups, slaughtered at 177 days of age

Trait	Genetic group				*P*-value*
	DL	LL	PL	PP	
FBW (Kg)	150.50 ± 9.06^a^	130.60 ± 8.77^b^	115.90 ± 7.02^c^	81.10 ± 7.31^d^	< 0.001
HCW (Kg)	122.00 ± 8.22^a^	106.70 ± 7.76^b^	93.50 ± 6.25^c^	64.50 ± 6.27^d^	< 0.001
CCW (Kg)	119.00 ± 8.53^a^	104.50 ± 7.68^b^	91.60 ± 6.28^c^	63.10 ± 6.23^d^	< 0.001
HCY (%)	81.00 ± 1.95^ab^	81.70 ± 0.95^a^	80.70 ± 0.83^ab^	79.50 ± 1.28^b^	0.029
CCY (%)	79.10 ± 2.21	80.00 ± 0.94	79.00 ± 0.93	77.80 ± 1.25	0.074
LMW (Kg)	28.10 ± 6.60^a^	25.10 ± 1.89^a^	17.20 ± 2.61^b^	10.30 ± 2.22^c^	< 0.001
FW (Kg)	22.40 ± 4.08^a^	19.50 ± 2.48^ab^	23.00 ± 2.69^a^	16.50 ± 3.15^b^	< 0.001
BNW (Kg)	9.10 ± 0.95^a^	7.38 ± 0.53^b^	6.49 ± 0.59^c^	4.72 ± 0.41^d^	< 0.001
LEA (cm²)	62.60 ± 6.85^a^	56.80 ± 8.11^a^	36.80 ± 9.56^b^	27.00 ± 5.19^b^	< 0.001
BT (mm)	22.50 ± 6.17^b^	20.90 ± 3.39^b^	36.40 ± 4.75^a^	32.00 ± 9.13^a^	< 0.001
LMP (%)	45.90 ± 10.60^a^	47.70 ± 2.28^a^	36.20 ± 3.10^b^	32.70 ± 5.95^b^	< 0.001
FP (%)	37.70 ± 6.19^b^	37.90 ± 2.53^b^	49.60 ± 3.82^a^	52.00 ± 5.85^a^	< 0.001
BNP (%)	15.00 ± 1.27	14.30 ± 1.06	14.20 ± 1.62	15.00 ± 0.82	0.395

FBW: final body weight; HCW: hot carcass weight; CCW: cold carcass weight; HCY: hot carcass yield; CCY: cold carcass yield; LMW: lean meat weight from left carcass; FW: fat weight from left carcass; BNW: bone weight from left carcass; LEA: loin eye area; BT: backfat thickness; LMP: lean meat percentage; FP: fat percentage; BNP: bone%. SD: standard deviation. *ANOVA P values

 The PP group also presented lower lean meat weight, fat weight (FW), and bone weight (BNW). However, when considering relative body weight, the fat and lean meat percentages of the PP group did not differ from the PL group. Similarly, the DL and LL groups did not differ in lean meat percentage (LMP) and fat percentage (FP). PP (52.00 ± 5.85%) and PL (49.60 ± 3.82%) presented higher fat percentages in comparison to DL (37.70 ± 6.19%) and LL (37.90 ± 2.53%). Bone percentages did not differ across all groups (Table 2).

By standardizing the data using percentages, it became evident that the main differences between genetic groups are concentrated in cuts of higher commercial value, such as ham, shoulder, and loin (Table 3). The PP group showed significantly lower yields of WH and ham (*P* < 0.05), while the DL and LL showed superior performance in loin and shoulder yields. For loin, the DL and LL groups had significant higher yields, whereas the PL group did not differ significantly from the PP group for any evaluated prime cut.

**Table 3:** Means and standard deviations of primal cut yields for barrows

Trait	Genetic group				*P*-value*
	DL	LL	PL	PP	
Head (%)	7.07 ± 0.59	6.81 ± 0.63	7.03 ± 0.43	7.70 ± 0.86	0.064
Sirloin (%)	7.38 ± 1.81^a^	6.36 ± 0.79^ab^	5.60 ± 1.31^ab^	5.24 ± 1.33^b^	0.024
WL (%)	24.30 ± 2.99	23.70 ± 1.33	23.90 ± 2.54	25.10 ± 3.50	0.742
Tenderloin (%)	1.52 ± 0.25	1.31 ± 0.17	1.37 ± 0.14	1.32 ± 0.29	0.216
WR (%)	17.00 ± 1.23	16.60 ± 1.42	16.80 ± 1.35	17.70 ± 1.60	0.828
Belly (%)	8.98 ± 2.38	6.63 ± 2.52	8.78 ± 1.46	8.79 ± 0.98	0.145
WS (%)	12.70 ± 1.46	14.20 ± 1.03	13.80 ± 1.08	14.20 ± 1.02	0.093
WH (%)	26.40 ± 1.67^a^	25.90 ± 1.11^a^	23.90 ± 1.06^b^	23.00 ± 1.61^b^	< 0.001
Feet (%)	2.31 ± 0.21	2.31 ± 0.10	2.16 ± 0.27	2.48 ± 0.32	0.132
Rib (%)	12.50 ± 2.48	12.90 ± 1.33	11.60 ± 0.29	11.50 ± 1.75	0.514
Ham (%)	21.20 ± 1.63^a^	20.40 ± 1.08^a^	16.80 ± 1.02^b^	15.60 ± 1.24^b^	< 0.001
Picnic shoulder (%)	9.83 ± 0.75^ab^	10.42 ± 0.76^a^	8.89 ± 0.93^b^	8.78 ± 0.74^b^	< 0.001
Loin (%)	15.40 ± 2.87^a^	15.20 ± 1.38^a^	12.20 ± 1.94^b^	11.20 ± 1.43^b^	< 0.001

WL: loin with tenderloin, sirloin, fat and skin; WR: rib with fat, skin and bone; WS: picnic shoulder with fat, skin and bone; WH: ham with fat, skin and bone. SD: standard deviation. *ANOVA P values

### Meat quality, collagen content and chemical composition

There were no statistical differences in pH45 values among the DL, PL, and LL groups, while LL and PL had lower pH45 than PP. Regarding pH24, the only statistical divergence occurred between the PP and LL groups (Table 4). All groups reached final pH values within the range considered ideal.

**Table 4:** Carcass pH means and standard deviations measured at 0, 45 min, and 24 h post-mortem

Trait	Genetic group				*P*-value*
	DL	LL	PL	PP	
pH0	6.55 ± 0.322^a^	6.15 ± 0.359^b^	6.26 ± 0.120^ab^	6.63 ± 0.263^a^	< 0.001
pH45	6.21 ± 0.227^ab^	6.00 ± 0.237^b^	6.03 ± 0.240^b^	6.36 ± 0.188^a^	0.013
pH24	5.80 ± 0.269^ab^	5.67 ± 0.137^b^	5.76 ± 0.281^ab^	6.03 ± 0.183^a^	0.053

pH0: pH at slaughter; pH45: pH at 45 min after slaughter; pH24: pH at 24 h postmortem. SD: standard deviation. *ANOVA P values

There was no significant effect of genetic group on most chemical composition variables in either muscle, with the exception of dry matter in the *Longissimus dorsi*, for which the PP group exhibited lower values than the DL and LL groups (Tables 5 and 6). Ether extract content, expressed on a dry matter basis, did not differ between the PP and PL groups and the commercial groups, despite the higher backfat thickness of PP and PL. When expressed on a natural matter basis, loin ether extract content was significantly affected by genetic group (*P* = 0.033); however, Tukey’s post-hoc test did not identify significant pairwise differences among the specific groups.

**Table 5:** Meat quality, chemical composition, and collagen content of *Longissimus dorsi*: means and standard deviations

Trait	Genetic group				*P*-value*
	DL	LL	PL	PP	
*Meat quality*					
Drip loss (%)	5.40 ± 2.63^a^	4.57 ± 2.49^ab^	3.65 ± 2.16^ab^	2.34 ± 0.75^b^	0.053
Shear force (Kgf)	2.49 ± 0.64	2.59 ± 0.37	2.70 ± 0.56	2.48 ± 0.55	0.795
*Color measurements*					
L*_LM_	57.90 ± 2.55^a^	56.70 ± 2.67^ab^	53.70 ± 4.16^b^	48.60 ± 2.87^c^	< 0.001
a*_LM_	7.81 ± 1.13	8.50 ± 1.11	8.32 ± 0.86	8.05 ± 0.77	0.413
b*_LM_	15.30 ± 0.97^a^	15.40 ± 1.24^a^	14.40 ± 2.03^a^	12.40 ± 0.99^b^	< 0.001
L*_F_	78.20 ± 2.13^ab^	77.50 ± 1.85^b^	79.80 ± 1.40^a^	79.50 ± 1.41^ab^	0.032
a*_F_	2.91 ± 0.59	3.48 ± 0.46	3.73 ± 0.64	3.54 ± 1.52	0.102
b*_F_	13.50 ± 0.70^b^	14.70 ± 1.46^a^	14.20 ± 0.81^ab^	13.60 ± 0.57^ab^	0.033
*Loss exudates*					
Defrost loss (%)	10.55 ± 3.33^a^	10.89 ± 2.22^a^	6.89 ± 2.36^b^	4.22 ± 2.92^b^	< 0.001
Cooking loss (%)	16.54 ± 9.22^a^	12.64 ± 2.68^ab^	10.64 ± 5.04^ab^	6.81 ± 1.90^b^	0.018
Total loss (%)	25.42 ± 8.41^a^	22.14 ± 3.34^ab^	16.73 ± 6.27^bca^	10.71 ± 4.21^c^	< 0.001
*Chemical composition*					
Dry matter (%)	33.70 ± 1.99^a^	33.80 ± 3.31^a^	31.40 ± 1.58^ab^	29.80 ± 1.88^b^	0.002
Mineral matter (%)	3.50 ± 0.89	4.21 ± 1.29	4.67 ± 1.57	4.54 ± 0.78	0.089
Crude protein (%)	57.30 ± 6.52	59.60 ± 10.88	63.50 ± 3.23	62.30 ± 5.99	0.182
Ether extract (%)	32.10 ± 4.62	27.90 ± 12.83	24.50 ± 4.19	23.70 ± 7.76	0.062
*Natural matter*					
Mineral matter (%)	1.17 ± 0.29	1.46 ± 0.43	1.48 ± 0.48	1.36 ± 0.28	0.229
Crude protein (%)	19.2 ± 1.16	19.7 ± 2.18	19.9 ± 1.10	18.5 ± 1.16	0.276
Ether extract (%)	10.88 ± 2.13	10.34 ± 5.61	7.65 ± 1.33	7.13 ± 2.59	0.033
*Collagen content*					
Total Collagen (mg/g)	9.07 ± 2.87	8.95 ± 2.34	8.18 ± 1.92	7.75 ± 2.60	0.646
Collagen solubility (%)	39.60 ± 4.72	42.60 ± 6.77	35.60 ± 7.26	44.10 ± 8.35	0.045

L*_LM_: L* parameter for lean meat; a*_LM_: a* parameter for lean meat; b*_LM_: b* parameter for lean meat; L*_F_: L* parameter for fat; a*_F_: a* parameter for fat; b*_F_: b* parameter for fat. SD: standard deviation. *ANOVA P values

**Table 6:** Meat quality, chemical composition, and collagen content of *Biceps femoris*: means and standard deviations

Trait	Genetic group				*P*-value*
	DL	LL	PL	PP	
*Meat quality*					
Drip loss (%)	4.35 ± 2.34	4.45 ± 3.29	2.81 ± 0.86	2.48 ± 0.81	0.130
Shear force (Kgf)	2.73 ± 0.83^b^	3.15 ± 0.83^ab^	3.96 ± 1.01^a^	3.06 ± 1.39^ab^	0.034
Color *measurements*					
L*_LM_	50.90 ± 3.82^a^	45.30 ± 2.39^b^	43.70 ± 2.48^bc^	40.50 ± 2.45^c^	< 0.001
a*_LM_	9.76 ± 0.72	10.35 ± 0.99	9.97 ± 0.77	9.55 ± 1.08	0.293
b*_LM_	14.36 ± 1.42^a^	12.29 ± 1.10^b^	11.44 ± 1.40^b^	9.15 ± 0.78^c^	< 0.001
L*_F_	75.60 ± 3.38	76.50 ± 2.89	75.20 ± 1.31	78.50 ± 3.11	0.293
a*_F_	2.84 ± 1.01	2.81 ± 0.61	3.39 ± 1.49	3.17 ± 0.42	0.697
b*_F_	13.50 ± 1.55	14.10 ± 1.22	14.00 ± 1.89	13.90 ± 0.57	0.887
*Loss exudates*					
Defrost loss (%)	7.94 ± 3.46	7.16 ± 3.36	6.58 ± 3.20	3.29 ± 14.6	0.073
Cooking loss (%)	17.10 ± 4.08	14.50 ± 4.89	14.50 ± 3.89	13.40 ± 6.62	0.335
Total loss (%)	23.70 ± 4.2	20.60 ± 6.07	20.10 ± 4.57	16.10 ± 14.7	0.077
*Chemical composition*					
Dry matter (%)	30.60 ± 3.62	29.40 ± 2.53	28.60 ± 1.63	29.50 ± 3.23	0.413
Mineral matter (%)	6.13 ± 2.71	7.11 ± 3.04	8.66 ± 2.65	8.42 ± 4.39	0.203
Crude protein (%)	57.70 ± 12.26	62.90 ± 7.42	67.20 ± 3.93	58.2 ± 7.92	0.063
Ether extract (%)	25.70 ± 10.02	22.40 ± 8.24	17.20 ± 4.27	26.30 ± 10.96	0.082
*Natural matter*					
Mineral matter (%)	1.82 ± 0.75	2.06 ± 0.81	2.48 ± 0.80	2.51 ± 1.46	0.267
Crude protein (%)	17.39 ± 2.93	18.36 ± 1.35	19.14 ± 0.93	16.99 ± 1.04	0.089
Ether extract (%)	8.17 ± 4.46	6.76 ± 3.09	4.95 ± 1.46	8.06 ± 4.03	0.135
*Collagen content*					
Total Collagen	7.13 ± 1.28^b^	9.61 ± 2.88^a^	7.51 ± 2.27^ab^	6.24 ± 1.63^b^	0.027
Collagen solubility (%)	21.50 ± 5.54	20.40 ± 3.35	20.80 ± 6.48	18.40 ± 5.90	0.791

L*_LM_: L* parameter for lean meat; a*_LM_: a* parameter for lean meat; b*_LM_: b* parameter for lean meat; L*_F_: L* parameter for fat; a*_F_: a* parameter for fat; b*_F_: b* parameter for fat. SD: standard deviation. *ANOVA P values

No differences were observed for loin collagen content and shear force (*P* > 0.05; Table 5). Loin collagen solubility was significantly affected by genetic group (*P* = 0.045); however, Tukey’s post-hoc test did not identify significant pairwise differences among the specific groups. The PP group showed lower L* and b* indices for the loin, characterizing a darker meat profile. For loin samples, the PP group showed lower water losses compared to the DL group. The PL group did not differ from the other groups regarding drip loss, cooking loss, and total loss.

Significant differences were observed in meat quality traits for the ham, specifically for defrost loss, total collagen content, shear force, and L^*^ and b^*^ color indices (Table 6). A greater force was required to shear the meat of the PL group when compared to the DL group, indicating that the DL group is more tender than the Piau crossbred. In contrast, the LL, DL and PP groups did not differ from each other. The DL group also exhibited higher L^*^ and b^*^ indices, indicating that its meat is lighter (greater intensities of yellow and luminosity). Darker meat, with significantly lower L* and b* values, was also observed in the PP ham.

Total collagen content was higher for LL than for PP and DL (Table 6), although collagen solubility did not differ significantly. None of the exudate loss measurements differed significantly among groups. Drip loss and color parameter a* also did not differ significantly among groups.

## Discussion

### Performance

According to Moreira et al. (2022), it is expected that the Piau breed (purebred or crossbred) exhibits lower daily gain, poorer feed conversion, and higher subcutaneous and intramuscular fat deposition than commercial lines. The lower performance observed for the PP group is consistent with prior literature suggesting that local breeds, lacking intensive selection for lean meat deposition, prioritize total fat deposition and maintenance over muscle protein synthesis. Since lipid synthesis requires significantly more dietary energy than muscle tissue deposition, the growth efficiency of these breeds is considerably lower than that of genotypes such as Duroc and Large White, which have been rigorously selected for rapid growth and optimized feed conversion.

The PL group demonstrated the effectiveness of crossbreeding with commercial breed to enhance performance. The higher performance of the PL group compared with PP may be explained by heterosis effects, combined with the contribution of the Large White breed’s genetic potential for growth. Although both are crossbred animals, there is a clear divergence between the DL and PL groups. The DL group combines two highly selected commercial lines, leveraging the terminal sire effect of the Duroc breed to accelerate growth and lean meat yield. In contrast, the PL group carries the genetic contribution of the Piau breed, a local breed characterized by slower growth and higher fat deposition, which worsens feed conversion.

As FC is a ratio, the absence of differences among PP, PL, and LL in F1 phase is explained by the fact that the Piau breed had lower feed intake and ADG compared with the LL group. On the other hand, PL did not differ from LL regarding ADG and ADFI. The similarity between PL and LL for ADG and ADFI may reflect a heterosis effect and the contribution of Large White for growth performance. The findings in this study are consistent with studies indicating that local breeds have lower growth efficiency compared to commercial breeds, such as Duroc and Large White, which are often selected for superior growth and feed conversion traits (Wood et al. 2004).

### Carcass traits

The absence of a significant difference in BT between the PP and PL groups is an interesting finding. It suggests that crossing the Piau breed with commercial lines (PL) improves carcass weight and performance without reducing subcutaneous fat deposition at the slaughter age. The maintenance of high BT in the PL group, despite its higher hot carcass weight compared to the purebred Piau, indicates that the genetic potential for fat depostition in the Piau breed remains even when crossed with lines selected for lean mass gain. Subcutaneous fat is associated with energy reserve and thermal insulation traits, while intramuscular fat (marbling) directly influences the tenderness, juiciness and sensory quality of meat (Poklukar et al. 2020). Breeds genetically selected for greater production efficiency, such as commercial pigs, tend to accumulate less subcutaneous fat and a greater proportion of lean muscle mass, in contrast to local breeds, which show greater deposition of fat (Serão et al. 2011; Poklukar et al. 2020). These inherent genetic differences in fat deposition directly contribute to variations in feed conversion, overall performance, and the ultimate composition of the carcass.

Crossbreeding Piau with Large White significantly increased cold and hot carcass weights in comparison to purebred Piau. This finding indicates a carcass weight advantage of PL over PP, which may have economic relevance given industry payment structures based on hot carcass weight. However, PL and PP exhibited lower carcass weight than DL and LL. These results align with the fact that the LL and DL groups underwent intense selection for meat production, which explains their higher carcass weights. It is also important to highlight that DL had significantly higher carcass weight than LL. This may reflect heterosis effects; however, purebred Duroc was not evaluated in this study to confirm this hypothesis.

The yield of the primal cuts was expressed as a percentage of cold carcass weight rather than absolute weight to mitigate the confounding effect of differences in slaughter weight between the genetic groups and ensure a fair comparison between genotypes. This methodological choice was necessary because significant differences in slaughter weights would have biased the results in favor of the heavier DL and LL, since these groups reached higher final body weights than the PP group, the use of absolute weights would mainly reflect differences in size rather than actual differences in tissue deposition and carcass modulation. By standardizing the data using percentages, it became evident that the main differences between genetic groups are concentrated in cuts of higher commercial value, such as ham, shoulder, and loin.

The lower yields of WH and ham in the PP group indicate that, even when size is taken into account, ham muscle development is less pronounced in the local breed. This pattern highlights the success of intensive selection in commercial breeds for the development of high-yield prime cuts and suggests that, although the crossbred (PL) improves overall carcass weight, it maintains a cut modulation more similar to the Piau breed for these specific prime cuts. This aligns with the findings of Mote and Rothschild (2020), who noted that improved pigs with high meat-gain potential tend to maintain a higher yield of lean cuts compared with non-selected pigs. Thus, the genetic selection of the commercial groups (DL and LL) likely explains their higher loin, picnic shoulder, and ham yields in comparison to PP.

In summary, carcass traits showed distinct patterns of growth and carcass composition among genotypes. While the DL and LL groups had higher slaughter weights and greater proportional development of high-value prime cuts, the PP group showed greater fat deposition. The PL represented an intermediate phenotype for carcass weight, while retaining backfat thickness and prime cut yield similar to that of the Piau breed. Different pig breeds exhibit varying carcass compositions, with improved breeds generally possessing greater meat content (Saikia et al. 2024). The use of percentage yields indicates that genetic background influences not only animal size but also the distribution of carcass value across cuts, favoring muscularity in improved breeds and adiposity in local and crossbred genotypes.

### Meat quality, collagen content and chemical composition

The pH pattern demonstrates how genetic background shapes *post-mortem* biochemical reactions. According to Matthews et al. (2022), maintaining high pH levels within the first 45 min post-exsanguination is a decisive factor for meat quality, as it prevents the thermal denaturation of myofibrillar proteins while the carcass still retains body heat. In the present study, the fact that no group presented critical pH values (remaining above 6.0) explains the total absence of PSE (Pale, Soft, and Exudative) meat. This stability suggests that, although the DL group has a higher growth potential, its initial glycolytic rate was sufficiently controlled to avoid compromising protein integrity.

The gradual and uniform pH decline correlates directly with the favorable results for color and water-holding capacity (WHC) found in the evaluated cuts. As discussed by Andrés-Bello et al. (2013), a slow decline in pH allows proteins to maintain their quaternary structure and electrical charges, preserving the intermyofibrillar space for water retention. The divergence in pH24 between PP and LL may reflect differences in muscle glycogen reserves at slaughter or in the density of muscle fiber types between these specific genotypes; nonetheless, all groups followed the standard physiological decline driven by anaerobic metabolism (Caldara et al. 2012).

With the exception of pH, all other meat quality traits were evaluated in both the *Longissimus dorsi* and *Biceps femoris* muscles. Most studies focus on the evaluation of loin samples; however, the ham has the highest salable meat content and is used for high-value products, such as cured ham. Thus, understanding the differences among genetic groups regarding ham meat quality is highly relevant. There was no significant effect of the genetic group on most of the chemical composition variables for either muscle, with the exception of dry matter in the *Longissimus dorsi*. The PP group exhibited lower dry matter than the DL and LL groups (Tables 5 and 6). Although the PP and PL groups exhibit higher backfat thickness (subcutaneous adiposity), their ether extract content did not differ from that of the commercial groups. This can be explained by the fact that ether extract was measured after removing the visible external fat. Consequently, the samples represent exclusively the intramuscular fat (IMF) content. Thus, the pronounced adiposity of the Piau breed did not translate into higher lipid concentrations within the muscle tissue compared to the commercial lines.

Interestingly, when expressed on a natural matter basis, ether extract content showed a significant overall effect of genetic group (*P* = 0.033), although Tukey’s post-hoc test did not detect significant differences between specific group pairs. This apparent discrepancy between the dry matter and natural matter expressions of the same variable likely reflects the influence of dry matter content itself, which differed significantly among genetic groups in the *Longissimus dorsi* (*P* = 0.002); since the natural matter basis incorporates the overall moisture content of the sample, differences in dry matter percentage between groups may introduce additional variation that is not evident when ether extract is expressed relative to dry matter alone.

No differences were observed for loin collagen content and shear force (*P* > 0.05). Although the Piau breed showed lower weight gain, which normally indicates a slower growth rate, this did not translate into changes on meat texture and tenderness. Although the overall ANOVA indicated a significant effect of genetic group on collagen solubility (*P* = 0.045), Tukey’s post-hoc test did not detect significant differences between specific group pairs, likely reflecting the reduced statistical power of pairwise comparisons given the limited and unequal sample sizes across groups (PP, *n* = 6). Together with the absence of significant differences in shear force, this suggests that the PP group maintains relatively stable collagen structures that were not impacted by its slower growth rate. Regarding color, the lower L^*^ and b^*^ indices in the PP group has a darker meat profile, probably associated with a more oxidative muscle metabolism and higher myoglobin content.

Meat pH is known to affect traits such as color, water-holding capacity, and tenderness (Jerez-Timaure et al. 2022; Razmaitė et al. 2024). High final pH values (pH24), often associated with local breeds, have been linked to lower cooking losses and greater juiciness (Razmaitė et al. 2024). The higher pH24 observed in the PP group compared with LL may therefore be associated with better water retention. For loin samples, the PP group showed lower total and defrost losses compared to the LL group. Also, PP exibithed lower water losses than DL. This superior Water Holding Capacity (WHC) in the purebred Piau breed suggests that its muscle proteins are highly effective at retaining moisture which may result in juicier product. The PL group, in turn, did not differ from the other groups regarding drip loss, cooking loss, and total loss. The high water retention observed in the Piau breed reflects greater capacity to retain moisture during processing, a trait relevant to further product oriented studies.

Significant differences in defrost loss, total collagen content, shear force, and L^*^ and b^*^ color indices were were observed for the ham. A greater force was required to shear the meat of the PL group when compared to the DL group, indicating that the DL group is more tender than the Piau crossbred. In contrast, the LL, DL and PP groups did not differ from each other. The DL group also exhibited higher L^*^ and b^*^ indices, indicating that its meat is lighter (greater intensities of yellow and luminosity), which, for the end consumer, makes the meat more attractive when compared to the PL, LL and PP meat. Darker meat was also observed in the PP ham, characterized by significant lower L^*^ and b^*^ values. This may be a disadvantage for this group, as consumers are accustomed to lighter-colored pork.

The variation in total collagen content without a corresponding change in collagen solubility indicates that differences in muscle texture are likely linked to connective tissue matrix density. Total collagen was higher for LL than PP and DL. This structural variation could impact tenderness, however, LL did not differ from the other groups in shear force. Unlike the results observed for the loin, none of the exudate loss measurements differed significantly among groups in the ham, although the DL group tended to show higher defrost loss than the PP group (*P* = 0.073; Table 6). The fact that parameters such as defrost loss, drip loss, and color parameter a* were not significant suggests that, for this specific muscle, suggests that, for this specific muscle, differences between the Piau breed and commercial lines (DL and LL) are less pronounced than observed in the Longissimus dorsi.

The differences observed between the comparison across groups for loin and ham highlight the influence of muscle type on the physicochemical and technological properties of pork, as well as the possible perception of the meat by consumers (Li et al. 2024). The *Longissimus dorsi*, commonly used in meat quality studies, has predominantly glycolytic fiber composition, which may be associated with lower meat quality, including higher toughness and lighter color (Kim et al. 2013; LeMaster et al. 2024). This muscle showed significant differences among genetic groups for variables such as dry matter content, drip loss, color parameters (L* and b*) and exudate loss, suggesting a greater influence of genetic group. In contrast, the *Biceps femoris* muscle, which has a higher proportion of oxidative fibers and plays a more prominent locomotor role, exhibited less variation among groups, with differences limited to shear force, defrost loss and total collagen content. These findings indicate that the *Biceps femoris* is less affected by genetic group.

It is worth noting that the number of Piau (PP) animals included in this study was relatively small compared to the other genetic groups, resulting in unequal group sizes. This reflects the fact that the Piau breed is maintained as a conservation herd with a limited population size, which restricted the number of viable matings available during the study period. Although the sample size was sufficient to detect significant differences between Piau and the other genetic groups for several traits, it may have limited the statistical power to detect more subtle differences, particularly for traits with high natural variation.

## Conclusion

This study highlights the importance of genetic group for the performance, carcass traits, and meat quality of pigs. The purebred Piau (PP) combined higher water-holding capacity with lower growth performance and hot carcass weight compared with the commercial breeds and crossbreeds. The Piau × Large White cross (PL) improved growth performance and hot carcass weight relative to PP while preserving the high subcutaneous fat deposition characteristic of the local breed.

The Duroc × Large White cross (DL) combined superior growth rates, feed conversion, and lean meat proportion with a lighter meat color, a trait commonly associated with consumer preference in commercial pork (citação, se tiver). No differences in intramuscular fat were observed among groups, contrary to the expectation that Piau and Duroc cross would present higher intramuscular fat than Large White purebred.

## Supplementary Information

Below is the link to the electronic supplementary material.

Supplementary Material 1

## Data Availability

The datasets analysed during the current study are available from the corresponding author on request.
