# Supplementary material for: Influence of Piau and commercial genetic groups on the performance, carcass traits, and meat quality of pigs
Source: Trop Anim Health Prod. 2026 Sep 21;58(8):547. doi: 10.1007/s11250-026-05335-9 (PMC13593715; doi:10.1007/s11250-026-05335-9)
Supplement: Supplementary file 1 [file 11250_2026_5335_MOESM1_ESM.docx]

**Supplementary material**

**Table 1** Chemical and nutritional composition of diets.

| Ingredient | Age of the animal (Phase) | | | |
| --- | --- | --- | --- | --- |
|  | 77 - 98 (G1) | 99 - 119 (G2) | 120 - 140 (F1) | 141 - 165 (F2) |
| Corn | 685.8160 | 755.5423 | 813.0868 | 861.8065 |
| Soybean meal | 259.2695 | 199.1132 | 147.4061 | 102.6110 |
| Soybean oil | 21.3895 | 16.8961 | 13.9342 | 11.9104 |
| Dicalcium phosphate | 14.1300 | 10.6003 | 8.9236 | 7.8628 |
| Limestone | 7.0182 | 5.9135 | 5.3683 | 5.0290 |
| Salt | 4.0579 | 4.0531 | 3.6723 | 3.5444 |
| L-Lysin | 3.4866 | 3.5025 | 3.5224 | 3.5129 |
| L-Threonine | 1.2832 | 1.1345 | 1.0031 | 0.8849 |
| L-Tryptophan | 0.2748 | 0.3057 | 0.3430 | 0.3635 |
| DL-Methionine | 1.2745 | 0.9388 | 0.7401 | 0.4747 |
| Minerals | 1.0000 | 1.0000 | 1.0000 | 1.0000 |
| Vitamins | 1.0000 | 1.0000 | 1.0000 | 1.0000 |
| Nutritional composition | | | | |
| NE (Kcal/Kg) | 2524.7350 | 2552.9715 | 2577.4997 | 2599.7202 |
| ME (Kcal/Kg) | 3350.0001 | 33500.0001 | 3350.0002 | 3350.0002 |
| CP (%) | 17.9756 | 15.6978 | 13.7277 | 12.0028 |
| Calcium (%) | 0.7221 | 0.5750 | 0.4970 | 0.4440 |
| Sodium (%) | 0.1800 | 0.1800 | 0.1650 | 0.1600 |
| Available phosphorus (%) | 0.3570 | 0.2833 | 0.2448 | 0.2187 |
| Digestible Lysin (%) | 1.0690 | 0.9270 | 0.8050 | 0.6970 |
| Digestible MET + CIST (%) | 0.6310 | 0.5470 | 0.4830 | 0.4180 |
| Digestible Methionine (%) | 0.3700 | 0.3122 | 0.2711 | 0.2261 |
| Digestible Threonine (%) | 0.6950 | 0.6030 | 0.5230 | 0.4530 |
| Digestible Tryptophan (%) | 0.2140 | 0.1850 | 0.1610 | 0.1390 |
| Digestible Valine (%) | 0.7380 | 0.6400 | 0.5550 | 0.4810 |
| NE: Net energy; ME: Metabolizable energy; CP: Crude protein and MET + CIST: Methionine + Cystine | | | | |
